# Peeling Back the Layers of Lymph Gland Structure and Regulation

**DOI:** 10.3390/ijms23147767

**Published:** 2022-07-14

**Authors:** Bayan Kharrat, Gábor Csordás, Viktor Honti

**Affiliations:** 1 *Drosophila* Blood Cell Differentiation Group, Institute of Genetics, Biological Research Centre, P.O. Box 521, H-6701 Szeged, Hungary; bayan.kharrat@brc.hu; 2 Faculty of Science and Informatics, Doctoral School of Biology, University of Szeged, P.O. Box 427, H-6720 Szeged, Hungary; 3 Lysosomal Degradation Research Group, Institute of Genetics, Biological Research Centre, P.O. Box 521, H-6701 Szeged, Hungary; csordas.gabor@brc.hu

**Keywords:** hematopoiesis, *Drosophila*, lymph gland, HSC, niche

## Abstract

During the past 60 years, the fruit fly, *Drosophila melanogaster*, has proven to be an excellent model to study the regulation of hematopoiesis. This is not only due to the evolutionarily conserved signalling pathways and transcription factors contributing to blood cell fate, but also to convergent evolution that led to functional similarities in distinct species. An example of convergence is the compartmentalization of blood cells, which ensures the quiescence of hematopoietic stem cells and allows for the rapid reaction of the immune system upon challenges. The lymph gland, a widely studied hematopoietic organ of the *Drosophila* larva, represents a microenvironment with similar features and functions to classical hematopoietic stem cell niches of vertebrates. Lymph gland studies were effectively supported by the unparalleled toolkit developed in *Drosophila*, which enabled the high-resolution investigation of the cellular composition and regulatory interaction networks of the lymph gland. In this review, we summarize how our understanding of lymph gland structure and hematopoietic cell-to-cell communication evolved during the past decades and compare their analogous features to those of the vertebrate hematopoietic stem cell niche.

## 1. Background—The *Drosophila* Model of Hematopoiesis

Hematopoiesis is the process through which hematopoietic stem cells (HSCs) differentiate to replace all types of blood cells throughout an organism’s life. During human embryogenesis, hematopoiesis starts in the aorta–gonad–mesonephros region and the yolk sac, moving to the foetal liver, spleen, and thymus (for T-cells) and ending in bone marrow, which becomes the main site of hematopoiesis after birth [1]. HSCs reside in a specialized microenvironment in the bone marrow, called the HSC niche, where they interact with other hematopoietic and non-hematopoietic cells. These interactions are essential for the regulation of their number and their ability to differentiate [2]. In *Drosophila*, as in humans, blood cell production occurs in two waves. The first embryonic wave, occurring in the head mesoderm, gives rise to macrophage-like blood cells that migrate throughout the embryo during its development, as well as non-migratory crystal cells [3,4,5,6]. The second hematopoietic wave starts in the dorsal mesoderm and produces immune cells, which remain clustered and form the precursor to a larval hematopoietic organ, the lymph gland [3,4,7].

Hemocytes, the blood cells of *Drosophila*, are similar in function to certain mammalian myeloid immune cells, while the lymphoid lineage is absent [8,9,10]. Hemocytes in the larval circulation were categorized into three types: plasmatocytes, crystal cells and lamellocytes [11,12,13,14]. Plasmatocytes are the most common cell type, accounting for 90–95% of circulating hemocytes. The functions of these small round cells are phagocytosis of microbes and apoptotic cells as well as secretion of antimicrobial peptides and extracellular matrix (ECM) proteins, the latter of which play an indispensable role in tissue damage repair [3,11,15,16]. Crystal cells comprise 2–5% of hemocytes and can be distinguished from plasmatocytes based on their morphology. These cells develop characteristic crystalline inclusions of prophenoloxidase enzymes in their cytoplasm, which are necessary in the melanization cascade and, thus, wound healing. Furthermore, crystal cells are crucial in the defence against pathogens in that they produce cytotoxic reactive oxygen species (ROS) as by-products of the melanization cascade [11,17,18]. Lamellocytes are typically absent from the circulation of healthy larvae, but rapidly appear upon immune induction or various stimuli, such as injury, wasp parasitization or the presence of tumours. These are large flat cells of variable shapes and sizes with either smooth edges or protruding cytoplasmic extensions. Notably, this cell type is required for the encapsulation response against parasitic wasp eggs, during which lamellocytes form a multilayered shell around the invader to restrict its access to hemolymph nutrients [5,17,19,20,21].

For several years, it was assumed that the three effector hemocyte classes differentiate from distinct progenitor cells. Recently, it was shown that plasmatocytes are more heterogeneous than they were previously believed; single-cell transcriptome data revealed several individual plasmatocyte clusters with diverse functions [22,23,24]. These experiments also underlined the plasticity of plasmatocytes, identifying a cluster capable of converting into lamellocytes via a process called transdifferentiation [5,22,23,24,25]. The ability of *Drosophila* immune cells to switch fates is analogous to mammalian immune cell transdifferentiation, as reviewed by Shen et al. (2004) [26], and highlights the adaptive capacity of immune cells across phyla.

Another important parallel with mammalian hematopoiesis is the fact that *Drosophila* larval immune cells form hematopoietic compartments, where hemocyte proliferation and differentiation take place. Larval hemocytes are found in three compartments: the circulation, the sessile hematopoietic compartment, in which hemocytes are located in pockets between the body wall muscles and the larval epidermis [5,20,27,28], and the previously mentioned lymph gland, a central compact hematopoietic organ attached to the dorsal vessel [17,29].

The conserved regulation of hematopoiesis, the functional similarity, the plasticity of blood cells and the presence of hematopoietic compartments render *Drosophila* an excellent model to study blood cell differentiation. Hence, the investigation of the fruit fly hematopoiesis has expanded considerably in the past decades, with particular focus on the central hematopoietic organ, the lymph gland.

## 2. The *Drosophila* Lymph Gland

### 2.1. Discovery and Early Characterization

The *Drosophila* lymph gland was first described in 1931 by Stark and Marshall as a lymphatic organ which acts as a reservoir for blood cells and phagocytes [30]. In 1955, El Shatoury characterized its structure and the cells it harbours more thoroughly. He described the lymph gland as a multi-lobed organ surrounding the heart, with the anterior pair of lobes (also called primary lobes) being the largest, with a triangular shape, and the other four pairs being smaller in size and showing a tubular morphology [29]. According to his description, all five pairs of lymph gland lobes show a similar structure, with three main types of cells: hexagons, platelets and spheroids. At that time, the hematopoietic role of the lymph gland was not described; instead, El Shatoury suggested that the lymph gland was an endocrine organ which would produce important factors regulating the differentiation of imaginal cells [29].

A later study on larvae with melanotic tumours found that the structure of the lymph gland is loose. The authors suggested that this is caused by the release of large cells—later identified as lamellocytes—either one by one or by the dissolution of the primary lobes [31]. This theory was further supported by the suggestion that circulating hemocytes in tumorous larvae are derived from the lymph gland based on electron microscopic and histochemical evidence [17]. Lymph gland cells were divided into two groups based on Rizki’s nomenclature of circulating hemocytes [32]: the first one included prohemocytes and proplasmatocytes, while the other one contained procrystal and crystal cells [17].

Prohemocytes were described as small round or polygonal cells without any cytoplasmic processes and a less dense cytoplasm than proplasmatocytes. According to the description, depending on their differentiation stage, these cells may contain a higher number of organelles and cytoplasmic processes. On the other hand, procrystal cells were distinguished by the presence of small cytoplasmic regions containing fibrils. These regions grow in both size and in number, and the fibrils ultimately form crystals as the cells differentiate into crystal cells. Crystal cells were seen only in the anterior lobes and in a much lower number than procrystal cells. It is worth noting that cells with intermediate phenotypes between prohemocytes and proplasmatocytes were also observed in the lymph gland, as well as cells in various phases of crystalline synthesis [17].

### 2.2. Functional Zones, Cell Types and Regulation

Although early reports characterized the gross structure of the lymph gland, it was not clear how this organ was homologous to hematopoietic niches in mammals. The advent of transgenic markers and new microscopy techniques led to the realization that the cells of this small hematopoietic organ are not as homogenous as initially suggested. In fact, based on molecular and structural characteristics, the primary lobes of third instar larval lymph glands can be divided into three functionally distinct regions: the posterior signalling centre (PSC), which emits signals to control progenitor maintenance, the medullary zone (MZ), containing hemocyte progenitors, and the cortical zone (CZ), populated by the following differentiated hemocytes: plasmatocytes, crystal cells and lamellocytes [33,34,35].

The ***posterior signalling centre*** represents a group of slowly dividing cells at the posterior tip of the primary lobes. Unlike the remaining lymph gland cells which originate from the first two thoracic segments, PSC cells are derived from a few Antennapedia (Antp) expressing cells in the third thoracic segment, maintaining their identity from the embryo to the larva. PSC cells do not differentiate into hemocyte progenitors or mature hemocytes; instead, they emit signals that control the balance between progenitor preservation and differentiation. As this non-autonomous regulation of hematopoiesis resembles stem cell niches in mammals, the PSC was named a hematopoietic niche in *Drosophila* [33,35,36,37,38].

Among the important signals produced from the PSC that control progenitor differentiation are the Decapentapelagic (Dpp) and Hedgehog (Hh) morphogens. Dpp is an earlier signal produced from embryonic stages onward, while Hh is synthesized only from the late first instar stage. Both Dpp and Hh are secreted from the PSC and bind to their receptors, Thickveins (Tkv) and Patched (Ptc), respectively, in the MZ to inhibit premature hemocyte precursor differentiation [36,39]. Serrate (Ser), the ligand for the Notch (N) pathway, is also produced by the PSC and plays a crucial role in specifying progenitor differentiation into crystal cells [33]. Moreover, PDGF- and VEGF-related factor 1 (Pvf1), a ligand for the Pvf/Pvr signalling pathway, is produced from the PSC and binds to its receptor in the CZ to initiate a backward signal that is essential for progenitor quiescence in the MZ [40]. To further highlight the orchestrating role of the PSC, it was found that it produces Spitz (Spi), the ligand of the EGFR signalling pathway, which is essential for the differentiation of lamellocytes after wasp infestation and oxidative stress in the niche [41,42].

It was suggested that the PSC delivers these signals into the MZ via filopodia-like cellular projections, which can reach progenitor cells that are not in direct contact with the signalling centre [36], although this remains to be conclusively demonstrated. 

While Antp is required for the determination of PSC identity, several additional factors are needed for its development and function. One such regulator downstream of Antp is the transcription factor Collier (Col), the *Drosophila* ortholog of the vertebrate Early B cell Factor (EBF), which is essential for both PSC function and maintenance. PSC cells are present in *col* mutant embryos but are abolished by the third instar larval stage, which suggests that Col is required for their maintenance but not specification. In parallel, knocking *col* down in larval PSC cells impairs their ability to prime lamellocyte differentiation following wasp infestation [35]. In addition to Antp and Col, PSC cells express other markers that can be used to identify them, such as Dally-like (Dlp), a regulator of the Dpp pathway, Daughters against dpp (Dad), a downstream effector of the Dpp pathway, and Unpaired3 (Upd3), one of the JAK/STAT ligands in *Drosophila* [34,43].

The size of the PSC is regulated by multiple signalling pathways and transcription factors, including Antp itself [36]. Among these regulators are Dpp and Wingless (Wg), which play antagonistic roles by functioning upstream to the *dmyc* proto-oncogene. Overexpression of *wg* or silencing *dpp* upregulates *dmyc* gene expression, which increases the size of the PSC. The opposite effect is seen when silencing *wg* or overexpressing *dpp* [43,44]. Moreover, PSC size regulation is also subject to systemic factors, for example, the insulin-like peptides (Dilps) secreted from the insulin-producing cells (IPCs) in the brain, which activate the insulin pathway in the PSC and increase its size [45].

These factors are crucial, as changes in the size of the PSC affect the differentiation of hemocyte progenitors in the lymph gland. Reducing the PSC size increases the differentiation rate of progenitors and depletes the MZ pool, while increasing the number of PSC cells expands the number of progenitors at the expense of differentiated hemocytes [36]. The phenomenon that niche cell number is correlated to hematopoietic progenitor maintenance is not unique to *Drosophila* but was also observed in mammalian HSC niches [46].

It is worth mentioning that recent studies uncovered a second hematopoietic niche besides the PSC, established by the interaction of MZ cells with the dorsal vessel [47,48]. This cardiac niche is characterized by the secretion of two signalling molecules, the glycoprotein Slit (Sli) and the FGF ligand Branchless (Bnl). Sli controls progenitor maintenance indirectly by negatively regulating the size of the PSC through the Dpp pathway [47]. In contrast, Bnl acts directly on the progenitors in the MZ by sustaining normal levels of intracellular calcium, thereby preserving their immature state [48]. Importantly, the *Drosophila* cardiac niche invokes comparisons to mammalian HSC niches, where the stem cells receive signals from the surrounding vascular system [47].

The ***medullary zone*** is the medial region of the primary lobes, containing prohemocytes, which give rise to the differentiated hemocytes located in the cortical zone. These cells are more tightly packed and less granular than CZ hemocytes. Within the primary lobes, the expression pattern of several factors is restricted to the MZ, such as Thioester-containing protein 4 (Tep4), which has a thus far uncharacterized function in the MZ and Domeless (Dome), the only receptor for the JAK/STAT pathway in *Drosophila*. The JAK/STAT pathway plays an important role in maintaining progenitors in the MZ. Silencing components of this pathway increases the rate of progenitor differentiation, while their overexpression increases the size of the MZ [49]. Interestingly, JAK/STAT pathway activity is lost from the MZ if the integrity of the PSC is compromised, further supporting the role of the PSC as a niche [38].

Moreover, cell–cell interaction between MZ progenitors also contributes to their regulation. The adhesion molecule E-cadherin (E-cad), an adherens junction protein, is expressed at a higher level in the MZ and can be used as a marker to distinguish cells in this zone. Loss of E-cad in the progenitors induces differentiation, while its overexpression keeps progenitors in their undifferentiated state [50]. This is similar to mammals, where E-cad is required for embryonic stem cell renewal and maintenance [51,52]. At the same time, gap junctions play an important role in the MZ by allowing the passage of calcium signals needed for their maintenance. Knocking down gap junction components, such as Innexin 4/Zero population growth (Zpg), disrupts calcium signalling and causes premature progenitor differentiation [53].

Importantly, certain factors that regulate progenitors via the PSC are also expressed cell-autonomously in MZ hemocytes. An example of this is Wg, which also functions in the MZ to suppress premature differentiation of progenitors [44]. Similarly, Col was found to be also expressed at a low level in a subpopulation of MZ cells that co-express the marker Tep4, called “core progenitors” [35,54,55,56]. Loss of Col in this subpopulation leads to loss of certain MZ markers and to increased differentiation of plasmatocytes and crystal cells, while its overexpression increases the size of the MZ [54,55,57]. In addition, downregulation of *col* in the MZ upon wasp infestation is essential for lamellocyte development, while its MZ-specific overexpression blocks lamellocyte differentiation [55].

Interestingly, MZ hemocytes can sense environmental signals and respond independently from the PSC. For example, nutritional signals lead to changes in the levels of Dilps, which activate the Insulin/mTOR pathway in the progenitors. Inhibition or overactivation of this pathway favours the differentiation of the three effector hemocyte types at the expense of progenitors, suggesting that progenitor maintenance requires balanced dietary intake [44,45,58]. Moreover, olfactory stimulation leads to the secretion of the neurotransmitter Gamma aminobutyric acid (GABA) from neurosecretory cells into the hemolymph. This activates metabotropic GABA_B_ receptors (GABA_B_R) in the MZ, as well as calcium signalling necessary for progenitor maintenance [59]. Olfactory dysfunction does not only cause hematopoietic progenitor loss but also interferes with the larval immune response to parasitic wasps, since odorant receptor-mutant larvae fail to produce a high number of lamellocytes after wasp infestation [59,60]. Gaseous signals also affect progenitor differentiation in the lymph gland. Low levels of carbon dioxide (CO_2_) and oxygen (O_2_) induce the secretion of Dilp6 from the fat body, which in turn activates the insulin pathway and increases the levels of *Ser* expression in the lymph gland, resulting in the differentiation of blood precursors into crystal cells [61]. In addition, progenitors can sense oxidative stress; in fact, high levels of reactive oxygen species (ROS) are required for their proper differentiation. Scavenging ROS molecules from MZ cells blocks progenitor differentiation, while further increasing ROS levels induces their differentiation and lamellocyte production by activating the Jun N-terminal kinase (JNK) pathway [62].

Recently, in addition to GABA, the neurotransmitter dopamine was found to be involved in hemocyte progenitor regulation in the lymph gland. Surprisingly, unlike GABA, dopamine is produced by both the MZ and the PSC and is sensed by MZ cells. Disrupting dopamine synthesis in the progenitors blocks their cell cycle progression and results in smaller lymph glands with bigger MZ, while blocking dopamine sensing in these cells affects only lymph gland size [63].

The ***cortical zone*** of the primary lymph gland lobes is populated by differentiated effectors. These are plasmatocytes and crystal cells under homeostatic conditions, accompanied by lamellocytes after immune induction [34]. The CZ begins to form in the second instar larva. The earliest maturation markers expressed in this zone are Hml and Pxn, followed by effector cell type-specific factors in the third instar stage [34]. The volume of the CZ increases continuously at the expense of MZ cells until metamorphosis, when progenitors start to differentiate massively, resulting in the loss of the vast majority of Dome positive hemocytes. Afterwards, the lymph gland disintegrates and releases all of its hemocyte content into the circulation, where these cells help in the phagocytosis of bacteria and cellular debris associated with tissue remodelling during pupariation [20,34,64].

Congruent with the cell types present, CZ cells express markers characteristic to mature hemocytes found in the circulation. Some of them are produced by all CZ hemocytes, such as the aforementioned Hml [65] and Pxn [66], while others are specific to the individual effector cell lineages, such as the phagocytosis receptors NimC1 and Eater expressed by plasmatocytes [67,68,69]. Crystal cells, on the other hand, express two Prophenoloxidase (PPO) enzymes, PPO1 and PPO2 [15,70,71], Lozenge (Lz), a transcription factor required for their specification [72], Sima, an ortholog to the mammalian hypoxia-inducible factor–α (Hif-α) needed for their survival [73], and Hindsight (Hnt), a mediator of the N pathway [74]. Finally, lamellocytes can be recognized by the expression of a surface antigen of unknown function named Atilla [16,21], the presence of alpha and beta transmembrane receptor integrin subunits β-PS and αPS4 [15], the expression of PPO3 enzyme [71,75] and the increased levels of Misshapen (Msn) and Puckered (Puc), components of the JNK pathway, which is involved in lamellocyte differentiation [15,28,76,77].

Interestingly, differentiated hemocytes in the CZ also communicate with MZ cells to control progenitor maintenance. The “equilibrium signal” becomes activated when the Pvf1 ligand produced from the PSC binds to its receptor, Pvr, in the CZ. This leads to the secretion of the Adenosine deaminase growth factor-A (Adgf-A) enzyme to the MZ, which reduces its levels of free adenosine and, in turn, inhibits the G protein-coupled adenosine receptor (AdoR). This stabilizes an active Hh signalling in the progenitors and further supports their maintenance [40]. A second regulatory mechanism acting upstream to JNK, Toll and Ras/EGFR pathways to suppress premature progenitor differentiation is mediated through the endosomal proteins Rab5 and Rab11 [78].

The basic zonal structure of the lymph gland consisting of niche-cells, progenitors and differentiated immune cells became a simplified and attractive model to study hematopoiesis. This can be attributed to both the functional similarities between the *Drosophila* and the mammalian hematopoietic niche and the sophisticated approach that *Drosophila* provides to identify and manipulate cells at the molecular level.

### 2.3. Re-Evaluating Zones and Cell Type Diversity

With the development of genetic tools, sequencing techniques and a growing number of research groups devoted to deciphering lymph gland hematopoiesis, it became evident that the accepted “three zone structure” of the primary lobes is more intricate, both regarding cell types and regulatory mechanisms. In line with this trend, the latest single-cell sequencing data proposed the presence of multiple cell populations with intermediate differentiation states between progenitors and plasmatocytes or crystal cells. Notably, these results also validated the early microscopic observations that suggested a gradual hemocyte differentiation in the lymph gland [17,79,80].

Since the description of hemocyte progenitors in the lymph gland, the question has arisen whether these cells, or a sub-population of them, are equivalent to hematopoietic stem cells. In 2010, Minakhina and Steward [81] reported that the lymph gland contains pluripotent precursor cells which undergo mitotic divisions before differentiation, thus being able to self-renew. These cells were reported to be in close contact with the PSC, and their maintenance was found to be dependent on Zfrp8, the homolog of PDCD2, which is enriched in hematopoietic stem cells in humans [81]. Following this, Krzemien et al. (2010) comprehensively investigated stem cell properties, such as slow self-renewal, asymmetric division and different nucleolar size, but could not confirm the presence of real HSCs in *Drosophila* [82]. In 2016, Dey et al. reported again the presence of HSCs in the first instar lymph gland near the dorsal vessel, which express *STAT92E* and *N* and are negative for the MZ marker Dome. With lineage tracing experiments, they discovered that these cells are the founders of all Dome positive progenitors in the lymph gland and that their self-renewal depends on Dpp signalling from the PSC, resembling how HSCs are dependent on the ortholog of Dpp (BMP) secreted by the surrounding mesenchyme [39,83,84]. This was confirmed later by single-cell sequencing studies, reporting the presence of a group of cells proposed as precursors to all lymph gland progenitors, expressing high levels of *STAT92E*, *N* and *Delta* (*Dl*), the gene encoding a N ligand, while not showing expression of the *Tep4* MZ marker. Since sequencing data did not prove that these cells are able to divide asymmetrically as bona fide stem cells, instead of calling these hemocytes HSCs, they were named Prehemocytes 1 (PH1). In addition, genes preferentially expressed in this cluster were identified, such as the Hippo pathway-related transcription factor *Scalloped (Sd)*, genes related to chromatin remodeling, such as *Enhancer of bithorax* (*E(bx)*), *jim* and *YL-1*, as well as genes related to the ecdysone signalling, such as the *Ecdysone receptor* (*EcR*) and *crooked legs* (*crol*) [79]. To understand the regulatory functions of these factors in hemocyte progenitors, further investigations are necessary.

The heterogeneity of MZ cells was recognized before single-cell experiments, when they were divided into three sub-populations based on their differential expression of cellular markers. These populations are core progenitors (cells positive for Tep4 and Dome), distal progenitors (cells positive for Dome and negative for Tep4 and Hml) and intermediate progenitors (cells positive for Dome, Hml but negative for Tep4) [44,56]. Core progenitors are located closer to the medial of the primary lobes, intermediate progenitors are proximal to the cortex and distal progenitors reside in between [56]. Intermediate progenitors are the most well-characterized sub-population so far, with recent publications suggesting that these cells form a distinct lymph gland zone, the “intermediate zone” (IZ) [79,80,85]. To specifically mark and genetically manipulate IZ hemocytes, a Split-GAL4 driver named *CHIZ-GAL4* was developed [85]. Unlike the rest of MZ cells, IZ cells are mostly pre-mitotic and are controlled by the Wg and the Ras pathways in an opposing manner, with Wg being required for their maintenance in an undifferentiated state and Ras promoting their differentiation [44,85]. It is worth noting that the term “intermediate progenitors” was first used in 2010 by Krzemien et al. to refer to a group of dispersed mitotic cells between MZ and CZ which are negative for markers of both zones [82]. Later on, the term evolved to describe the non-mitotic, double-positive cells discussed above. 

Interestingly, these sub-populations of MZ cells were found not only to differ in their expression of molecular markers but also to be functionally distinct. For example, the activity of the N pathway is required in core progenitors for their maintenance, while it is needed in distal progenitors for their specification into crystal cells at the expense of plasmatocytes. For this reason, silencing components of the N pathway in core progenitors leads to a reduction in their number and to the differentiation of both plasmatocytes and crystal cells, while silencing them in distal progenitors suppresses the differentiation of crystal cells and promotes the differentiation of plasmatocytes [56].

Recently, benefiting from single-cell RNA-sequencing techniques, two studies investigated the heterogeneity of lymph gland cells in the larva. These reports came to the conclusion that hemocytes in the classical lymph gland zones are more diverse than they were believed to be earlier. This diversity also suggests that there is no linear path for hemocyte development but rather multiple routes that lead to effector cell differentiation [79,80].

Single-cell transcriptomic experiments not only refined our understanding of “classical” cell types, such as prohemocytes, plasmatocytes, crystal cells, lamellocytes and PSC cells, but also identified two additional lymph gland hemocyte clusters. One of these clusters was named “GST-rich”, based on the finding that the cells of this cluster express *glutathione-S-transferase* transcripts at a higher level than other cells of the lymph gland, suggesting that they play a role in ROS-mediated stress response [79]. Two additional genes with yet unknown function, *CG3397* and *CG18547*, were also validated as cluster markers. By comparing the differentially expressed genes, this cluster is very similar to cluster “X”, identified by Girard et al. (2021), which predominantly expresses genes involved in mitosis, cell cycle control and DNA damage response [80], including *string* (*stg*) and *Myb*. It contains around 2000 cells, which can be divided into three subclusters based on their differential expression of MZ and CZ markers, suggesting that they represent cells that are mitotically active in the different zones of the lymph gland [80]. Of note, this also draws an interesting parallel to the previously mentioned initial description of intermediate progenitor cells by Krzemien et al. in 2010, which were defined as mitotically active hemocytes dispersed between the MZ and CZ [82]. The cells in the second cluster were named adipohemocytes, referring to their production of enzymes involved in lipid metabolism. These cells contain lipid droplets in their cytoplasm, similar to how they were described in other insect species [86,87], however, their function in the *Drosophila* lymph gland remains to be investigated [79].

Agreeing with Blanco-Obregon et al. (2020) [56], the cells of the medullary zone were classified into two clusters: MZ1, which corresponds to core progenitors, and MZ2, which is most similar to distal progenitors [80]. Sequencing data showed that both MZ1 and MZ2 express Dome and Tep4 markers, with MZ1 cells expressing both at a higher level than MZ2. It was also shown that MZ1 but not MZ2 hemocytes produce factors, such as Col and Dlp, at a low level. Since these factors are highly expressed in the PSC, it is plausible that these cells play a signalling role as well [80]. Interestingly, the *Ultrabithorax* (*Ubx*) homeotic gene, which was previously found to be expressed in the posterior lobes [88], is also active in the MZ1 cluster, which suggests that core progenitors are similar to posterior lobe progenitors [80]. The expression of downstream effectors of Toll and Imd pathways, such as Cecropin genes (*CecA1*, *CecA2*), is enriched in MZ2, hinting that these cells may be involved in the response of the lymph gland to microbial immune challenges. The transcriptomic signatures also show higher levels of glycolytic gene expression in MZ1 cells compared to MZ2, implying a different energetic requirement for fulfilling their respective functions [80].

Similarly to the MZ, the CZ cells were also divided into three main clusters: proplasmatocyte (proPL), plasmatocyte (PL) and crystal cell (CC). proPLs are the ancestors of PLs; they express plasmatocyte markers at a lower level than PL and appear earlier in the lymph gland. Although proPLs were plotted between the IZ cell and PL clusters, their transcriptomic signature is different from the other two subsets. One example is genes involved in the equilibrium signals, such as *Pvr* and *STAT92E*, the loss of which, in proPLs but not in IZ or PLs, causes increased differentiation, suggesting that these cells are the main players in the feedback signalling to the progenitors. Another notable difference is that while the ECM protein-coding genes *viking* (*vkg*) and *Col4a1* (*Cg25C*) are highly expressed in all three clusters, genes involved in secretion of these proteins, *sec23* and *Rab1*, are enriched only in PLs, suggesting that only mature plasmatocytes are able to secrete ECM proteins. Unlike IZ and proPL cells, a small subset of PLs shows a high expression level of *E-cad*. As E-cad is enriched in MZ cells, this population might differentiate directly from MZ progenitors without passing through proPL or IZ stages [80].

Single-cell sequencing also allowed the division of crystal cells into two subclusters: immature (iCCs) and mature crystal cells (mCCs). These can be easily distinguished, since mCCs show a higher expression of previously identified crystal cell markers (*lz*, *PPO1,2* and *hnt*) and are also bigger and more granular than iCCs. In addition, mCCs and not iCCs have large punctae of N, Sima and Numb, a protein that plays a role in N endocytosis [80]. The internalization of the N protein with the help of Sima and Numb activates the N pathway in a ligand-independent manner and plays a role in the maturation and survival of mCCs [73,80]. Another feature that distinguishes mCCs from iCCs is that they undergo endocycling, resulting in bigger nuclei [74,80,82]. Gene expression related to stress and unfolded protein response is also upregulated in the CC cluster, confirming previous reports that crystal cells sense ROS produced after immune induction and, in turn, are activated to release PPOs, which play a role in melanin production needed for wound closure, as well as the encapsulation and killing of foreign invaders [79,80,89,90]. Curiously, a small subset of CCs was found to express *NimC1* in addition to *lz*, suggesting that this group of crystal cells transdifferentiates directly from mature plasmatocytes, which was demonstrated in earlier publications [74,80,91].

Using single-cell sequencing to analyse the changes to the cellular composition of the lymph gland after wasp infestation, it was uncovered that core progenitors decrease in number while intermediate progenitors and GST-rich populations expand and lamellocytes differentiate [79]. In addition, agreeing with previous studies [35,92], the number of crystal cells is reduced in wasp-infested lymph glands. Although the transcriptomic profiles within the individual clusters were not altered significantly after wasp infestation, some progenitor populations and plasmatocytes expressed higher levels of cell cycle genes [79]. 

Lamellocytes differentiated after wasp infestation could be divided into two subpopulations representing immature and mature lamellocytes, with lamellocyte markers *Msn*, *attila*, *PPO3* and *methuselah-like 4* (*mthl4*) (a newly identified marker with yet unknown function in hematopoiesis) being expressed at a higher level in mature lamellocytes [79]. Taking all of this together, single-cell sequencing data suggest that immune induction primes progenitors and plasmatocytes to differentiate into lamellocytes at the expense of crystal cells.

**Figure 1:**
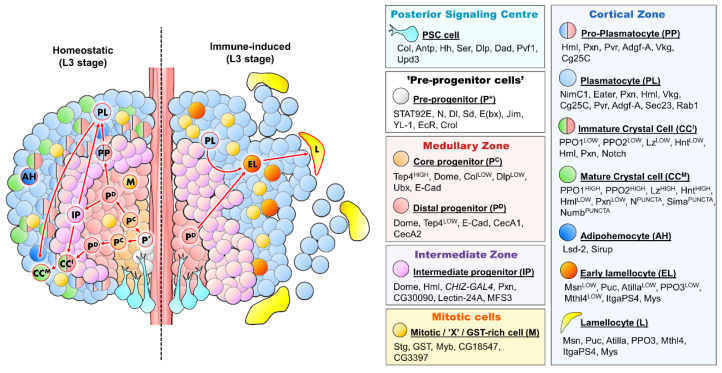
The main functional zones and cell types of the *Drosophila* lymph gland. The primary lobe of a third instar larva is shown under homeostatic conditions (**left** half) and following immune induction (**right** half). The different hemocyte lineages are indicated with red arrows. The individual cell types shown on the figure are defined on the right panels, along with their respective marker molecules and the zones they reside in.

In conclusion, single-cell sequencing experiments revealed that the lymph gland is a much more intricate hematopoietic organ than it was initially described to be and that it contains hemocytes in various stages of differentiation (Figure 1). It is also important to mention that single-cell sequencing of hematopoietic stem and progenitor cell (HSPC) niches in mammals has come to similar conclusions, suggesting that HSCs undergo a continuous process of maturation, rather than a discrete step-by-step development, with the presence of HSPCs in diverse transitionary states with different gene expression profiles [93,94,95].

## 3. Hematopoietic Regulatory Mechanisms—*Drosophila* and Mammals

The researchers who first identified the *Drosophila* lymph gland could not anticipate that this small organ renders strong homology on the molecular level with HSC niches in mammals. Similar to hemocyte progenitors in the lymph gland, HSCs are sensitive to changes in the microenvironment surrounding them, and they respond either by increasing their self-renewal, entering quiescence or differentiating. HSCs in the bone marrow (BM) are controlled by factors secreted from their surrounding niche cells, such as osteoblasts and stromal cells. One of these conserved factors is the secreted protein Slit, which plays a role, albeit different, in both mammalian and *Drosophila* hematopoietic niches. In *Drosophila*, the Sli ligand secreted from the vascular niche positively regulates the size of the PSC, which in turn suppresses progenitor differentiation in the MZ of the lymph gland [47]. In mice, the Slit3 ligand produced from the stroma of the bone marrow regulates the homing of HSPCs to the bone marrow [96] and the expression of the Slit receptor Robo4 by the vascular endothelium promotes HSC migration into the BM and restricts exiting into the blood [97].

Similarly to how the N pathway maintains core progenitors in the *Drosophila* lymph gland and plays a role in the binary decision between plasmatocyte/crystal cell fate in the distal progenitors [56,74,98], the mammalian N pathway functions in HSC self-renewal and differentiation, both in vitro and in vivo, and is involved in lymphoid vs. myeloid lineage commitment in vivo [99,100,101].

Another example of conserved hematopoietic regulation across taxa is the JAK/STAT pathway, which is required for HSC homeostasis in mice. HSCs are depleted in both *JAK1* and *JAK2* knockout mice [102,103], a phenotype similar to the reduction in the progenitor amount in the *Drosophila* lymph gland after the loss of JAK/STAT signalling activity in these cells [49].

The Hh pathway functions in a similar manner in both mammalian and fly hematopoiesis. In humans, Sonic hedgehog (Shh) was found to promote the proliferation of primitive hematopoietic progenitors [104], reminiscent of the role of its *Drosophila* counterpart, where it maintains MZ progenitors [36]. However, in the lymph gland, the downstream mechanisms of Hh activity related to progenitor maintenance remain to be conclusively defined.

The FGF pathway also plays a role in hemocyte precursor maintenance in both mammals and *Drosophila*. In *Drosophila*, the FGF ligand Bnl, secreted from the vascular niche, directly increases the number of progenitors in the lymph gland [48], while in mammals, FGFs stimulate HSC proliferation either directly, by binding to HSC receptors, or by activating receptors on stromal cells in the hematopoietic niche, inducing proliferation and secretion of FGFs and other growth factors that enhance HSC maintenance [105].

Similar to *Drosophila*, where Wg and Dpp signalling regulates progenitor maintenance, both in a cell-autonomous fashion by restricting progenitor differentiation and a non-cell-autonomous manner by regulating the size of the PSC [36,39,43,44], their mammalian orthologs, Wnt and BMP-4, regulate both HSCs and components of the niche, such as osteoblasts [46,100,106,107]. The Wnt pathway was shown to be essential for the maintenance of HSCs and the osteoblast niche cells in the bone marrow, as well as for suppressing premature differentiation of HSCs [100,107]. On the other hand, BMP-4 controls the number of HSCs directly in a dose-dependent manner: higher concentrations of BMP-4 lead to HSC maintenance, while lower concentrations of BMP-4 promote HSC division and maturation. BMP-4 also indirectly influences hematopoiesis by positively regulating the number of osteoblast niche cells in mice, which in turn increases the number of HSCs [46,106].

Interestingly, a role for neurotransmitters in controlling HSCs and hemocyte progenitors was described both in *Drosophila* and in mammals. Blocking GABA receptor (GABAR1) in mice reduces HSC numbers, reconstitution capacity and their ability to differentiate to platelets and B-cells. GABAR1 activation induces platelet formation in human cell lines and mice and also improves the engraftment ability of umbilical cord-derived human HSCs [108,109]. In *Drosophila*, silencing the GABA_B_ receptor leads to the depletion of hematopoietic precursors in the lymph gland, but this is due to enhanced differentiation, unlike in mammals, where abrogating GABA signalling interferes with HSC differentiation [59,108,109]. The neurotransmitter dopamine was found to regulate both *Drosophila* and mammalian hematopoiesis. Blocking dopamine sensing in mammalian HSCs reduces their number and interferes with their function [110]. This is similar to how the inhibition of dopamine signalling in *Drosophila* hemocyte progenitors hinders their proliferation, differentiation and their response to immune challenges [63].

Yet another regulatory mechanism that shows similarities between fly and mammalian hematopoiesis is cell-cell contact between immune cell precursors, which is essential for HSC maintenance. Blocking gap junctions between stromal cells and HSCs affects HSC growth and proliferation in vitro, while knocking down Connexin-43, a gap junction protein in mice, interferes with HSC proliferation and regeneration ability after chemotherapy [111,112]. In the *Drosophila* lymph gland, gap junctions are necessary for proper calcium signalling, and hence, MZ maintenance [53]. Moreover, although the role of the adhesion molecule E-cad in hematopoietic progenitor maintenance in *Drosophila* is well established [50,113], the function of another cadherin (N-cad) in the bone marrow is still controversial. A few reports have suggested that N-cad functions in anchoring the HSCs to the osteoblast niche cells and that higher levels of N-cad increase HSC self-renewal, while lower levels promote differentiation [46,114,115]. In contrast, later reports have disagreed with these results and found that knocking down N-cad in mice has no effect on the hematopoietic niche, HSC maintenance or function, leaving the role of N-cad in HSCs in question [116,117,118].

Oxidative stress can also be sensed by HSCs and hemocyte progenitors. Low levels of ROS are essential for keeping HSCs in the bone marrow and hemocyte precursors in the lymph gland undifferentiated, while high ROS levels induce their differentiation [62,119]. Interestingly, in both systems, ROS was found to reduce the levels of the respective cell adhesion molecules in blood cell precursors and HSCs, which in turn triggers their differentiation [113,120]. Similarly, HSCs can sense nutritional signals via the insulin/mTOR pathway. Overactivation of the insulin pathway leads to HSC depletion as well as to uncontrolled differentiation, ultimately resulting in myeloproliferative disorder (MPD) and leukemia [121,122], while depletion of insulin growth factor-1 (IGF-1) was also shown to induce HSC differentiation [123]. These findings suggest that HSCs are very sensitive to changes in nutritional input and that both abnormally high and low levels of IGFs disrupt their quiescence. This is similar to *Drosophila*, where both overactivation and inhibition of the insulin pathway lead to progenitor loss and increased rate of differentiation [45,58].

**Figure 2:**
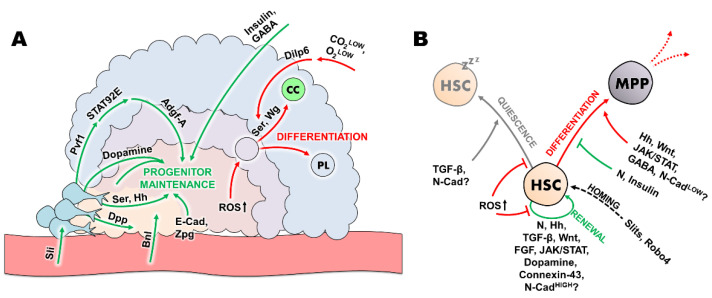
Hematopoiesis in the *Drosophila* lymph gland and in mammals is controlled by analogous mechanisms. (**A**) The schematic representation of a third instar *Drosophila* lymph gland demonstrates how hematopoiesis is controlled in the different zones. Green arrows indicate factors and mechanisms that promote progenitor maintenance, while red arrows indicate paths of differentiation into effector cells. CC: crystal cell, PL: plasmatocyte (**B**) The basic behaviours of the HSCs are indicated: homing (dashed), quiescence (grey), HSC renewal (green) and differentiation (red). The corresponding factors are shown next to the arrows. HSC: hematopoietic stem cell, MPP: multipotent progenitor.

Taken together, more and more studies highlight how the essential mechanisms controlling hematopoiesis in the fruit fly and mammalian HSC niches are maintained among species (Figure 2).

## 4. Perspectives

Our understanding of the *Drosophila* central hematopoietic organ, the lymph gland, has progressed at a rapid pace due to the advanced genetic arsenal available in the fruit fly. During this process, striking analogies with the mammalian hematopoietic stem cell niches were also uncovered. This made the *Drosophila* model an excellent tool to discover fundamental mechanisms that govern immune cell formation, stem cell-niche communication, transdifferentiation and immuno-metabolic crosstalk.

However, several questions remain unanswered. The recent single-cell sequencing studies provided a myriad of factors potentially involved in the regulation of lymph gland hemocyte sub-populations, which need to be followed up. Genetically tracing the identified hemocyte progenitor and lineage-committed (proplasmatocyte, immature crystal cell) clusters will also shed light on the origin of the different classes of mature hemocytes in normal and immune induced lymph glands, while revealing the possible routes of transdifferentiation. In addition, the lymph gland is very sensitive to various external stimuli (nutrition, odours, etc.), where our understanding of the exact mechanisms controlling progenitor maintenance vs. differentiation, or how these inputs are integrated, remains lacking. Finally, the lymph gland represents a rare opportunity to study how differentiating immune cells communicate with each other, with their progenitors and fully matured descendants in a hematopoietic tissue to orchestrate their differentiation under homeostatic conditions or in response to immune challenge. Exploring these avenues of research will no doubt contribute largely to our knowledge on the basics of blood cell differentiation in a hematopoietic niche.

## Data Availability

Not applicable.
